# Restrictive blood transfusion strategies and associated infection in orthopedic patients: a meta-analysis of 8 randomized controlled trials

**DOI:** 10.1038/srep13421

**Published:** 2015-08-26

**Authors:** Zhaowei Teng, Yun Zhu, Yugang Liu, Guojun Wei, Shuangneng Wang, Shaoliang Du, Xiguang Zhang

**Affiliations:** 1 Department of Orthopedic Surgery, The People’s Hospital of Yuxi City, The 6th Affiliated Hospital of Kunming Medical University, 21 Nieer Road, Yuxi 653100, Yunan, China; 2 Department of Nephrology, The People’s Hospital of Yuxi City, The 6th Affiliated Hospital of Kunming Medical University, 21 Nieer Road, Yuxi 653100, Yunan, China; 3 Department of Orthopedics of the Affiliated Hospital of Hebei University of Engineering, China; 4 Department of Orthopedics of the Second Affiliated Hospital of Harbin Medical University, China

## Abstract

This study sought to evaluate whether restrictive blood transfusion strategies are associated with a risk of infection in orthopedic patients by conducting a meta-analysis of randomized controlled trials (RCTs). RCTs with restrictive versus liberal red blood cell (RBC) transfusion strategies were identified by searching Medline, Embase, the Cochrane Central Register of Controlled Trials and the Cochrane Database of Systematic Reviews from their inception to December 2014. Eight RCTs with infections as outcomes were included in the final analysis. According to the Jadad scale, all studies were considered to be of high quality. The pooled risk ratio [RR] for the association between transfusion strategy and infection was 0.65 (95% CI, 0.47–0.91; p = 0.012), and the number of patients needed to treat to avoid an infection using a restrictive transfusion strategy was 62. No heterogeneity was observed. The sensitivity analysis indicated unstable results, and no significant publication bias was observed. This meta-analysis of RCTs demonstrates that restrictive transfusion strategies in orthopedic patients result in a significant reduction in infections compared with more liberal strategies.

Approximately 85 million units of red blood cells (RBCs) are transfused annually worldwide^1^, and more than 15 million RBC units are transfused in the United States^2^. Blood transfusion is commonly administered to elderly orthopedic surgical patients^3^,^4^, and subsequent transfusion-related infections frequently occur^5^.

Many studies^6^,^7^,^8^,^9^ have examined restrictive blood transfusion strategies in an attempt to decrease the number of transfusions, and the results show that although the number of transfused RBC units can be effectively reduced, the infectious events are not significantly decreased. A previous meta-analysis that evaluated restrictive transfusions ranging from 7 to 10 g/dL found that such treatment significantly reduced in-hospital mortality, though the effect on infection was only marginally significant (p = 0.046)^10^. Parker^6^ reported that restrictive blood transfusion strategies increase the risk of wound infections, whereas Rohde^11^ reported that restrictive blood transfusion strategies significantly reduce infection. Additional scientific evidence has subsequently been published, particularly in patients undergoing orthopedic surgery. Thus, it remains controversial whether restrictive blood transfusion strategies versus liberal blood transfusion strategies may reduce infectious events.

Restrictive blood transfusion strategies are widely used for older patients undergoing orthopedic surgery. However, to our knowledge, no specific meta-analysis of the association between infection risk and restrictive blood transfusion strategies has been conducted to date. Therefore, we performed a meta-analysis with the purpose of assessing the infection risk among orthopedic patients who had been subjected to restrictive transfusion strategies.

## Results

### Search results

**Figure 1:**
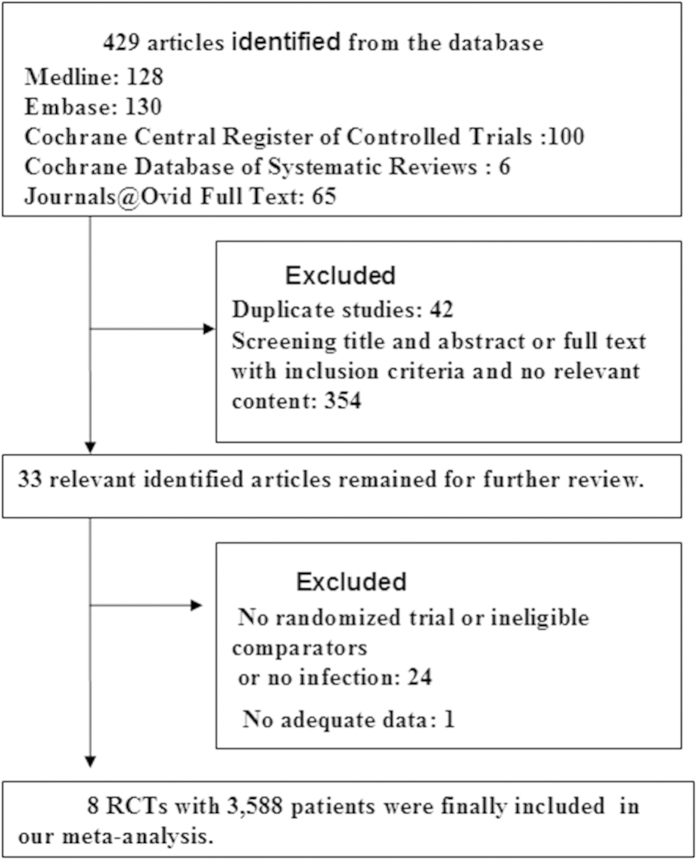


We identified 429 potential citations (128 from Medline; 130 from Embase; 100 from the Cochrane Central Register of Controlled Trials Databases; 6 from the Cochrane Database of Systematic Reviews and 65 from Journals@Ovid Full Text) for studies comparing a restrictive blood transfusion strategy and a liberal transfusion strategy for the treatment of orthopedic patients. After screening the title and abstract or by further reviewing full-text articles, 8 RCTs with infection as an outcome were ultimately identified. A total of 3,588 patients were included in the analysis (Fig. 1).

One trial was conducted in Denmark, 1 in the Netherlands, 1 in Canada, 1 in China, 1 in England, and 3 in facilities spanning multiple countries (United States, Scotland and Canada). The general characteristics of the 8 studies are summarized in Table 1, the infection outcome definition for the studies are summarized in Supplementary Table S1, and the quality scores for the studies are summarized in Supplementary Table S2 in the supplementary material. In these trials, the hemoglobin threshold ranged from 6.4 g/dL to 9.7 g/dL in the restrictive groups and from 8.0 g/dL to 10.0 g/dL in the liberal groups. Baseline hemoglobin levels were comparable between the two groups (Supplementary Table S3). Patients in the restrictive groups received fewer RBC units than those in the liberal groups (Supplementary Table S3 in supplementary material). The studies included were all of high quality (Jadad score ≥ 3.0).

### Meta-analyses

**Figure 2:**
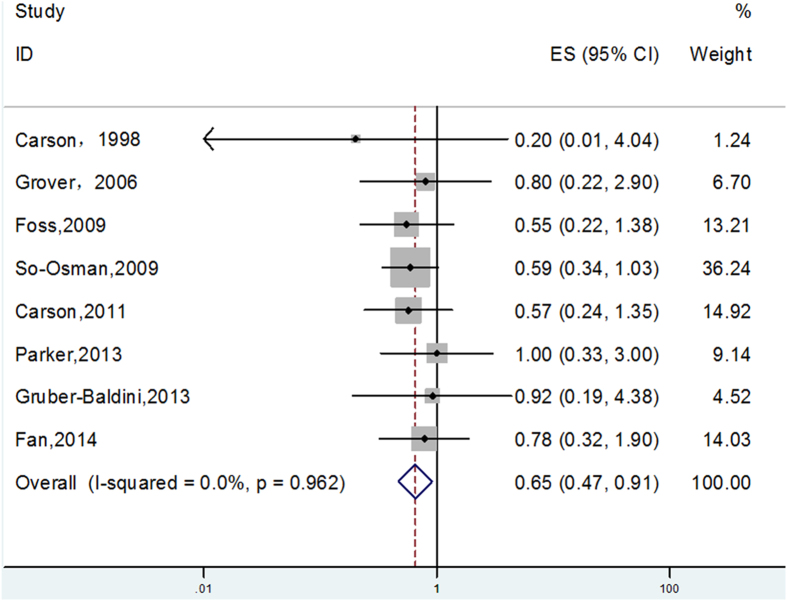


Eight studies with 3,588 patients provided information about infection. The overall pooled risk ratio for the association between transfusion strategy and infection was 0.65 (95% CI, 0.47–0.91; P = 0.012), as shown in the forest plot presented in Fig. 2. No heterogeneity was observed (P = 0.962, I^2^ = 0.0%).

We also conducted meta-analyses for wound infection and pneumonia. Of the 8 trials, 4 provided data on both wound infection and pneumonia, 1 provided data on wound infection only, and 1 provided data on pneumonia only. Restriction of the meta-analysis to the data on pneumonia yielded a pooled RR of 0.55 (95%CI, 0.24–1.25; P = 0.150) (Supplementary Fig. S1), whereas restriction of the meta-analysis to the data on wound infection yielded a pooled RR of 0.66 (95%CI, 0.35–1.24; P = 0.196) (Supplementary Fig. S2). Publication bias was not evident according to Begg’s test (P = 0.71) or the Harbord test (P = 0.905; 95%CI, −1.59–1.44) (Supplementary Figs S3 and S4).

**Figure 3:**
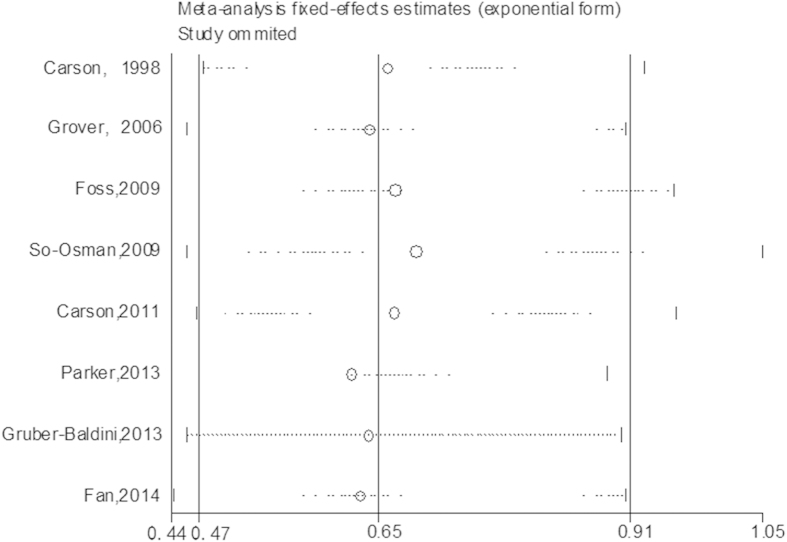


Sensitivity analysis was performed to evaluate the robustness of our investigation by individually omitting all studies from the pool. The patients in one trial^12^ received leukocyte-depleted RBCs, and after omitting this study, the pooled RR of infection was 0.69 (95%CI, 0.46–1.05; P = 0.082) (Fig. 3 and Supplementary Fig. S5). The cumulative statistical data also revealed that the combined risk ratios for the association between transfusion strategy and infection were not influenced by any other individual study (Fig. 3). In addition, one trial^8^ was performed in China, and after omitting this study, the combined RR of infection was 0.63 (95%CI, 0.44–0.91; P = 0.013).

The NNT with a restrictive transfusion strategy to prevent all infections was 62, and the number of avoided infections per 1,000 patients was 16.13.

## Discussion

This meta-analysis of RCTs was performed to describe the infection risk following restrictive RBC transfusion strategies compared to liberal RBC transfusion strategies in orthopedic patients. Our meta-analysis demonstrates that restrictive RBC transfusion strategies are associated with a 35% decrease in infection risk. The NNT to avoid infection using a restrictive transfusion strategy was 61. These findings are comparable to those of other recent meta-analyses^11^,^13^. When the data were restricted to wound infection or pneumonia, no statistically significant results were found. The findings in Rohde’s study^11^ showed that restrictive RBC transfusion strategies were associated with a 30% decrease in infection risk in orthopedic patients; however, our findings include more recent evidence with data from three additional trials. One study^9^ included the number of infections instead of the number of patients with infection; in our meta-analysis, the number of infections was used for this particular trial. However, Rohde’s study only extracted data on chest infection events (2 in the restrictive group and 3 in the liberal group), which may have led to inaccurate pooled results. In addition, Rohde’s study did not perform sensitivity analysis by omitting each study, especially omitting the trial which utilized leukocyte-depleted RBCs. In Salpeter’s study^13^, a meta-analysis of three RCTs showed that a restrictive hemoglobin transfusion trigger of <7 g/dL resulted in reduced in-hospital bacterial infections compared with a more liberal strategy; however, no data for orthopedic patients were provided. Therefore, although Rohde and his colleagues first described the relationship between restrictive blood transfusion strategy and infection risk in orthopedic patients, to the best of our knowledge, the findings of the present study are more thorough and robust.

Preoperative anemia is common in aged patients who have undergone major orthopedic surgery, and RBC transfusion is a common strategy used to treat anemia, particularly for patients showing symptoms or low hemoglobin concentrations^7^,^14^. Some previous studies^15^,^16^ performed RBC transfusions to increase oxygen-carrying capacity; however, despite increasing oxygen content, oxygen delivery was not increased. Moreover, to our knowledge, there is no trial evidence to date showing that blood transfusions significantly improve oxygen delivery. As transfusion-related adverse events are rather common and transfusion may affect infection risk by altering immune function^5^,^17^, decreasing blood transfusion may be beneficial for patients in some cases. Previous original studies^12^,^14^,^18^,^19^ reported that restrictive transfusion strategies could effectively decrease the number of units transfused, and one study^4^ reported that the number of units of RBCs transfused under a liberal transfusion strategy was 2.9 times greater than that under a restrictive transfusion strategy; nonetheless, the long-term risk of mortality due to infection was similar for the two transfusion strategies. In all of the studies included in our meta-analysis, it was also demonstrated that the number of transfused units was significantly reduced under a restrictive strategy; although no significant decrease in the risk of infection was found, the trend toward a decline was apparent in each of these studies. In the present meta-analysis, we observed that restrictive blood transfusion strategies could significantly decrease the risk of transfusion-associated infection. With regard to wound infection and pneumonia, although significantly decreased risks were not found, the decreasing trend was notable. Bernard *et al.*^20^ reported that the transfusion of 2 units of RBCs significantly increased the risk of pneumonia and surgical-site infection in general surgery patients. Carson *et al.*^21^ performed a retrospective cohort study of 9,598 consecutive hip fracture patients who underwent surgical repair, and the findings demonstrated that transfusion significantly increased both pneumonia and serious bacterial infections, which also included wound infections. Moreover, the cost of hospitalization was $14,000 greater for patients with serious infection than for patients without infection.

Publication bias was not observed in our meta-analysis. According to the sensitivity analysis, the combined results were unstable. One previous study^22^ reported that the transfusion of leukocyte-depleted RBCs could significantly decrease postoperative infections; after we omitted the trial^12^ using leukocyte-depleted RBCs, we found that the pooled results were unstable, which indicated that our results were likely due to differences in the utilization of leukoreduced products across randomized groups. The combined results after omitting the other trials were stable and robust according to the sensitivity analysis. We also obtained the same robust results after we omitted the trial^8^ conducted in Asia. However, it remains unknown whether associated infection risks are related to ethnicity, and more studies, particularly in non-Western populations, are required. One major strength of the current study was that all of the included reports adopted a randomized controlled design, and all were of good quality. Additionally, no heterogeneity was found in our analysis. The outcomes thus appear relevant to current practice.

Despite the advantages of this systematic review, there are some limitations. First, although we searched all RCTs describing the association between restrictive blood transfusion and infection, eligible studies were restricted to those published in the English language. In addition, the number of patients in some of the included studies was relatively small, implying that some studies may have led to underpowered results. Thus, larger-scale RCTs are needed, and our meta-analysis findings should be interpreted with caution. Second, studies with nonsignificant results, particularly those reporting the absence of an effect, may not be published because they are rejected by journals or because the investigators are unwilling to submit them for publication. Thus, the pooled results may be overestimated. Third, in this meta-analysis, the reporting of infectious outcomes varied across the included studies; in some studies, certain types of infections were listed, whereas in others, only one or two specific types of infections were reported. Fourth, in our meta-analysis, the number of infections was used, possibly double-counting patients who may have had multiple infections. Fifth, we identified no RCTs evaluating lower hemoglobin transfusion triggers for orthopedic patients, such as a level of <7 g/dL, which indicates that further studies are required. Sixth, as the included studies have different criteria for restrictive and liberal strategies, more studies with the same criteria are needed. Finally, we state that the quality of RCTs could be improved to include the greater use of blinding and more comprehensive ascertainment of all infections using standardized definitions.

In summary, we conducted a meta-analysis of RCTs and found that a restrictive transfusion strategy resulted in a significant reduction in infections compared with a more liberal strategy in orthopedic patients. However, larger scale and well-designed RCTs are still needed to aid clinicians in choosing an optimal transfusion strategy for patients undergoing orthopedic surgery.

## Methods

### Search strategy and data sources

We searched Medline (from 1946 to December 2, 2014), Embase (from 1947 to December 2, 2014), the Cochrane Central Register of Controlled Trials (October 2014) and the Cochrane Database of Systematic Reviews (from 2005 to October 2014) for randomized controlled trials (RCTs) describing the study requirements listed below. There were no restrictions regarding language or type of publication. We also searched the bibliographies of relevant articles to identify any additional studies. The last search was conducted on November 24, 2014. The detailed protocol design, as used previously by Rohde^11^, is shown in Supplementary Table S4.

### Study selection

Studies were considered eligible if they met all of the following criteria: (1) presented original data from an RCT; (2) used two comparator groups in which one group received a restrictive RBC transfusion strategy and the other received a liberal RBC transfusion strategy; (3) included orthopedic patients as the study participants; (4) reported infections as outcomes; and (5) had adequate data to be pooled for the analysis. If the data were duplicated or if the population was analyzed in more than one study, we included only the study with the largest sample size and the most comprehensive outcome evaluation.

### Data extraction and quality assessment

Two teams of independent investigators (TZW and LYG, ZY and WGJ) independently evaluated the eligibility of the studies retrieved from the databases based on the selection criteria. These two teams independently extracted the following data: the first author’s name, year of publication, patients’ ages, sample size, hemoglobin thresholds, and infectious outcomes. Any disagreements were resolved either by discussion or consultation with the corresponding co-author (ZXG). The assessment of methodological quality was based on the Jadad scale scoring system^23^, in which the maximum score is 5. We defined low quality as a Jadad score <3.0 and high quality as a score ≥3.0.

### Statistical analyses

We calculated the risk ratios and 95% confidence intervals (CIs) for each study using the DerSimonian and Laird random-effects model^24^. One study reported the hazard ratio (HR), which was considered to approximate the risk ratio of infection. We computed the pooled RR and 95% CI for any infection in all studies based on the calculated RRs and 95% CIs. Additionally, we also pooled the risk ratio of pneumonia and wound infection for the studies that provided adequate data. Cochran Q and I^2^ statistics were used to evaluate statistical heterogeneity^25^. When the P value was <0.1 and the I^2^ value was >50%, the data were considered to be heterogeneous, and a random-effects model (DerSimonian and Laird method) was applied to estimate the overall summary effect sizes. A fixed-effects model^24^,^26^ was used when no heterogeneity was present in the included studies. We calculated the number needed to treat (NNT) by using the risk of the event in the control group and the complement of relative risk (RR)^27^. To assess the stability of our results, a sensitivity analysis (by excluding each single study in turn) was conducted to estimate the influence of individual studies on the pooled result. We used the Harbord test^28^ and Begg’s test (rank correlation method)^29^ to assess potential publication bias.

## Additional Information

**How to cite this article**: Teng, Z. *et al.* Restrictive blood transfusion strategies and associated infection in orthopedic patients: a meta-analysis of 8 randomized controlled trials. *Sci. Rep.* **5**, 13421; doi: 10.1038/srep13421 (2015).

## Supplementary Material

Supplementary Information

## Figures and Tables

**Table 1:** Characteristics of the 9 RCTs included in the final analysis of transfusion strategies and infection risk.

Study, year	Age (years)	Country	Surgery	**Transfusion threshold**		RCT Size	Infection	**No. of Infections**			
				**R**	**L**			**Events(R)**	**Total(R)**	**Events(L)**	**total(L)**
Carson^18^	82.3 ± 9.5	US and Scotland	hip fracture repair	Hb < 8.0 g/dL in the absence of symptoms or symptomatic anemia	Hb < 10.0 g/dL	84	Pneumonia	0	42	2	42
Grover^9^	≥55	southeast England	elective lower limb joint replacement	Hb < 8.0 g/dL, maintenance range, 8.0–9.5 g/dL	Hb < 10.0 g/dL, maintenance range, 10.0–12.0 g/dL	218	Chest infection	2	109	3	109
							Wound infection	2	109	2	109
Foss^19^	≥65	Denmark	hip fracture repair	Hb < 8.0 g/dL	Hb < 10.0 g/dL	120	pneumonia	1	60	2	60
							Wound infection	0	60	3	60
							all infections	6	60	11	60
So-Osman^12^	≥18	Dutch	hip or knee replacement	threshold range, 6.4–9.7 g/dL	Varied by hospital, age and condition of patients, symptoms and time	619	Infections	18	299	31	304
Carson^14^	≥50	US and Canada	hip fracture repair	Symptomatic anemia or if Hb < 8.0 g/dl	Hb < 10.0 g/dL	2016	Wound infection	8	1007	14	1005
Parker^6^	≥60	Canada	hip fracture surgery	8.0–9.5 g/dL and symptomatic anemia	8.0–9.5 g/dL	200	Pneumonia	2	100	5	100
							Wound infection	3	100	1	100
							All infections	6	100	6	100
Gruber-Baldini^7^	≥50	US and Canada	hip fracture repair	symptoms or ≤ 8 g/dL	≤ 10 g/dL	139	Infection	3	72	3	66
Fan^8^	>65	China	hip replacement	Symptomatic anemia or if Hb < 8.0 g/dl, maintenance range, 8.0–10 g/dL	maintenance ≥ 10 g/dL	192	Pneumonia	3	94	3	92
							Wound infection	2	94	3	92
							All infections	8	94	10	92

Note: R represents restrictive blood transfusion strategies; L represents liberal blood transfusion strategies.
