# Supplementary material for: Restrictive blood transfusion strategies and associated infection in orthopedic patients: a meta-analysis of 8 randomized controlled trials
Source: Sci Rep. 2015 Aug 26;5:13421. doi: 10.1038/srep13421 (PMC4549631; doi:10.1038/srep13421)
Supplement: Supplementary Information [file srep13421-s1.pdf]

Supplementary Information

Restrictive blood transfusion strategies and associated infection in orthopedic patients: a meta-analysis of 8 randomized controlled trials

Zhao-wei Teng[1*¶], Yun Zhu[2¶], Yu-gang Liu[3], Guo-jun Wei[4], Shuang-neng Wang[1], Shaoliang Du[1], Xi-guang Zhang[1*]

[1]Department of Orthopedic Surgery, The People's Hospital of Yuxi City, The 6th Affiliated Hospital of Kunming Medical University, 21 Nieer Road, Yuxi 653100, Yunan, China.

[2]Department of Nephrology, The People's Hospital of Yuxi City, The 6th Affiliated Hospital of Kunming Medical University, 21 Nieer Road, Yuxi 653100, Yunan, China.

[3]Department of Orthopedics of the Affiliated Hospital of Hebei University of Engineering, China.

[4]Department of Orthopedics of the Second Affiliated Hospital of Harbin Medical University, China.

*Corresponding authors

Email: tengzhaowei2003@163.com (TZW) or gwkzxg@163.com (ZXG)

¶Zhaowei Teng and Yun Zhu are co-first authors.

Supplementary Table S1: Infection outcome definition of the 8 RCTs included in the final analysis.

Study, year	Definition
Carson, 1998	Used a modified Centers for Disease Control and Prevention (CDC) case definition of pneumonia: chest radiograph with new or progressive infiltrate, consolidation, or cavitation and any of the following: new onset of purulent sputum or change in character of sputum or the isolation of the organism from blood culture, transtracheal aspirate, bronchial brushings, or biopsy. Did not consider a patient with rales and purulent sputum to have pneumonia, and did not use pleural effusion in the chest radiograph definition.
Grover, 2006	New infections requiring antibiotic therapy
Foss, 2009	Any infectious complication such as pneumonia, sepsis and wound infection
So-Osman, 2009	The CDC criteria according to Horan's study (Horan TC, Gaynes RP, Martone WJ, Jarvis WR, Grace Emori T (1992) CDC definitions of nosocomial surgical site infections, 1992: A modification of CDC definitions of surgical wound infections. American Journal of Infection Control 20: 271-274.)
Carson, 2011	Wound infection; new onset purulent sputum; chest radiograph with new or progressive infiltrate
Parker, 2013	Any infectious complication such as pneumonia, superficial wound infection, deep wound infection and septicemia with septic shock
Gruber-Baldini, 2013	Any infections
Fan, 2014	Any infectious complication such as pneumonia, superficial wound infection and urinary tract infection

Supplementary Table S2: Methodological quality of studies included in the final analysis, based on the Jadad scale.

RCTs	Randomization 2			Blinding 2			An account of all patients 1	Total score
	1 point if randomization is mentioned	1 additional point if the method of randomization is appropriate	Deduct 1 point if the method of randomization is inappropriate (minimum 0)	1 point if blinding is mentioned	1 additional point if the method of blinding is appropriate	Deduct 1 point if the method of blinding is inappropriate (minimum 0)	The fate of all patients in the trial is known. If there are no data, the reason is stated	
Carson, 1998	1	0	0	1	0	0	1	3
Grover, 2006	1	0	0	1	0	0	1	3
Foss, 2009	1	1	0	1	1	0	1	5
So-Osman, 2009	1	0	0	1	1	0	1	4
Carson, 2011	1	1	0	1	1	0	1	5

Parker, 2013	1	1	0	1	1	0	1	5
Gruber-Baldini,2013	1	1	0	1	0	0	1	4
Fan, 2014	1	1	0	1	1	0	1	5

Supplementary Table S3: RBC transfused and baseline hemoglobin in the 8 RCTs.

Study, year	RBC transfused (R)	RBC transfused (L)	Baseline hemoglobin(R)	Baseline hemoglobin(L)
Carson, 1998	0 (Median) (IQR O-2) units; 19 (45.2%) patients received RBC transfusion	2 (median) (IQR 1-2); 41 (97.6%) patients received RBC transfusion	(9.1±0.6) g/dL	(9.1±0.6) g/dl
Grover, 2006	0 (Median) (Range O-5) units; 37 (34%) patients were transfused a total of 89 red cell units	0 (Median) (Range O-10) units; 46 (43%) patients received a total of 119 units	(13.1±1.22) g/dL	(13.6±1.22) g/dL
Foss, 2009	1 (Median) (IQR 1-2) units; 22 (37%) patients received transfusions	2 (Median) (IQR 1-2) units; 44 (74%) patients received transfusions	Not available but graphed	Not available but graphed
So-Osman, 2009	0.78 (Mean) ±(SD1.4); 105 (35%) patients received RBC transfusion	0.86 (Mean) ±(SD 1.6) 93 (31%) patients received RBC transfusion	(13.7±1.4) g/dL	(13.7±1.4) g/dL
Carson, 2011	0 (Median) (IQR O-1) units; 413 (41%) patients were transfused a total of 652 units	2 (Median) (IQR 1-2) units; 970 (97%) patients were transfused a total of 1866 units	(11.3±1.5) g/dL	(11.3±1.5) g/dL

Parker, 2013	No patient received a blood transfusion	All patients received a blood transfusion, with a mean of 1.9 units	11.8 g/dL	11.5 g/dL
Gruber-Baldini,2013	0 (Median) units; 33 (45.8%) patients were transfused a total of 53 units	2 (Median) units; 63 (95.4%) patients were transfused a total of 115 units	(11.9±1.7) g/dL	(11.9±1.3) g/dL
Fan, 2014	41(43.6%) patients received a blood transfusion	52 (56.5%) patients received a blood transfusion. More units transfused (p=0.03)	(12.0±1.1) g/dL	(11.8±1.2) g/dL

Note: R represents restrictive blood transfusion strategies; L represents liberal blood transfusion strategies.

Supplementary Table S4: Search strategy for meta-analysis designed by Rohde.

1. *Blood Transfusion/
2. ((Red blood cell* or RBC) adj3 (therap* or transfus*)).mp.
3. 1 or 2
4. exp Reference Standards/
5. standards.fs.
6. methods.fs.
7. 4 or 5 or 6
8. 3 and 7
9. ((H?emoglobin or h?emocrit or HB or HCT) adj5 (polic*or practic* or protocol* or trigger* or threshold*or indicator* or strateg* or criteri* or standard*)).mp.
10. (transfus* adj5 (polic*or practic* or protocol* or trigger* or threshold*or indicator* or strateg* or criteri* or standard*)).mp.
11. ((Red blood cell* or RBC) adj5 (polic*or practic* or protocol* or trigger* or threshold*or indicator* or strateg* or criteri* or standard*)).mp.
12. (transfus* adj5 (restrict* or liberal*)).mp.
13. ((blood or transfus*) adj3 (management or program*)).mp.

14. 8 or 9 or 10 or 11 or 12 or 13
15. randomi?ed.ab,ti.
16. randomized controlled trial.pt.
17. controlled clinical trial.pt.
18. placebo.ab.
19. clinical trials as topic.sh.
20. randomly.ab.
21. trial.ti.
22. 15 or 16 or 17 or 18 or 19 or 20 or 21<br>(animals not (humans and animals)).sh.
23. (animals not (humans and animals)).sh.
24. 22 not 23
25. 14 and 24
26. (hip or knee or orthopedic or fracture).ab,ti.

27. 25 and 26

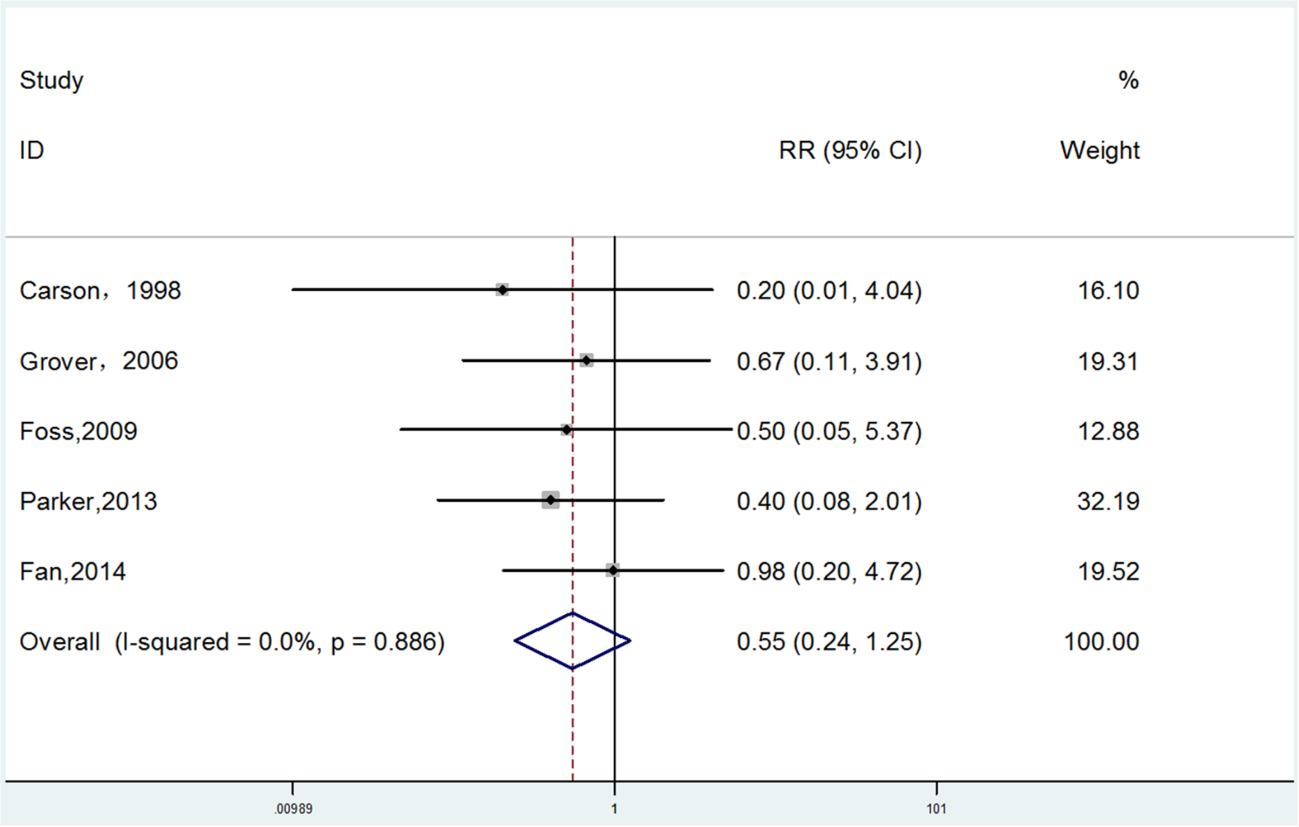

Supplementary Figure S1: Forest plot for pneumonia risk according to 5 RCTs in a fixed-effects model meta-analysis.

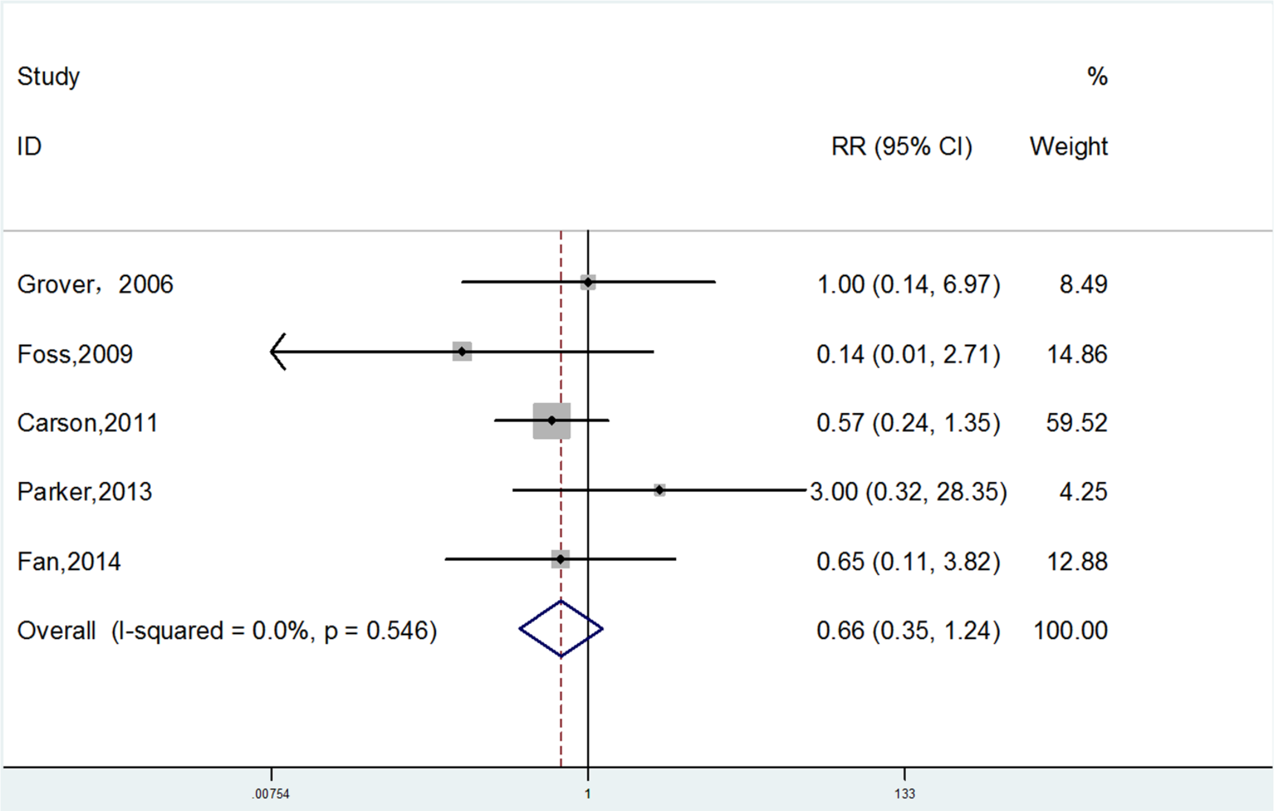

Supplementary Figure S2: Forest plot for wound infection risk according to 5 RCTs in a fixed-effects model meta-analysis.

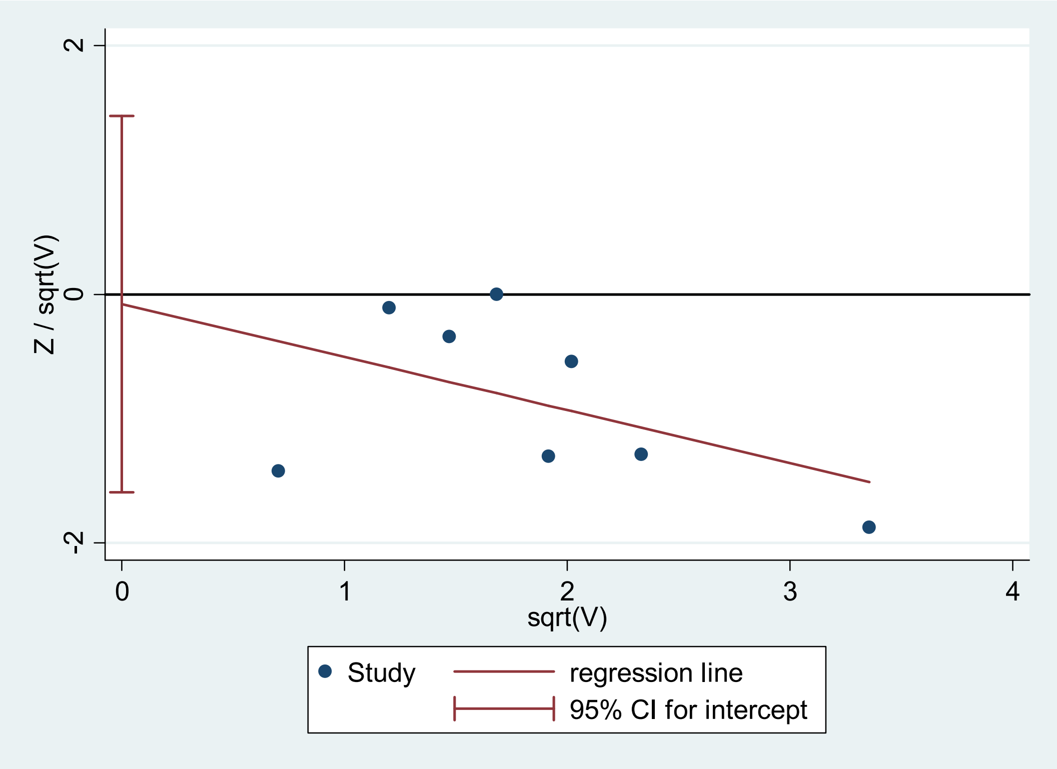

Supplementary Figure S3: Harbord publication bias plot of the 8 RCTs.

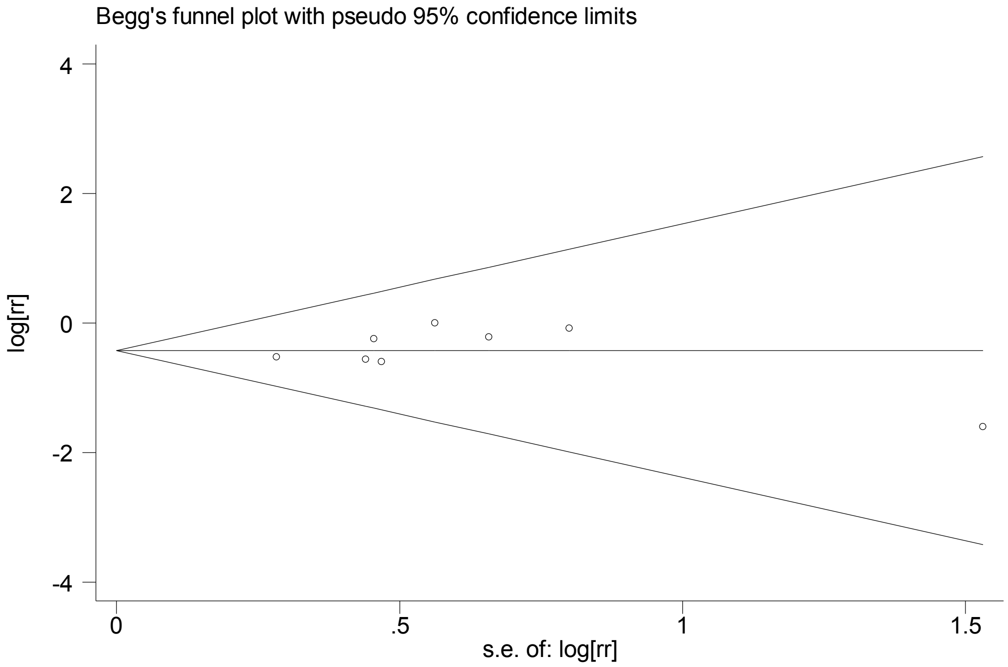

Supplementary Figure S4: Begg's funnel plot of the 8 RCTs.

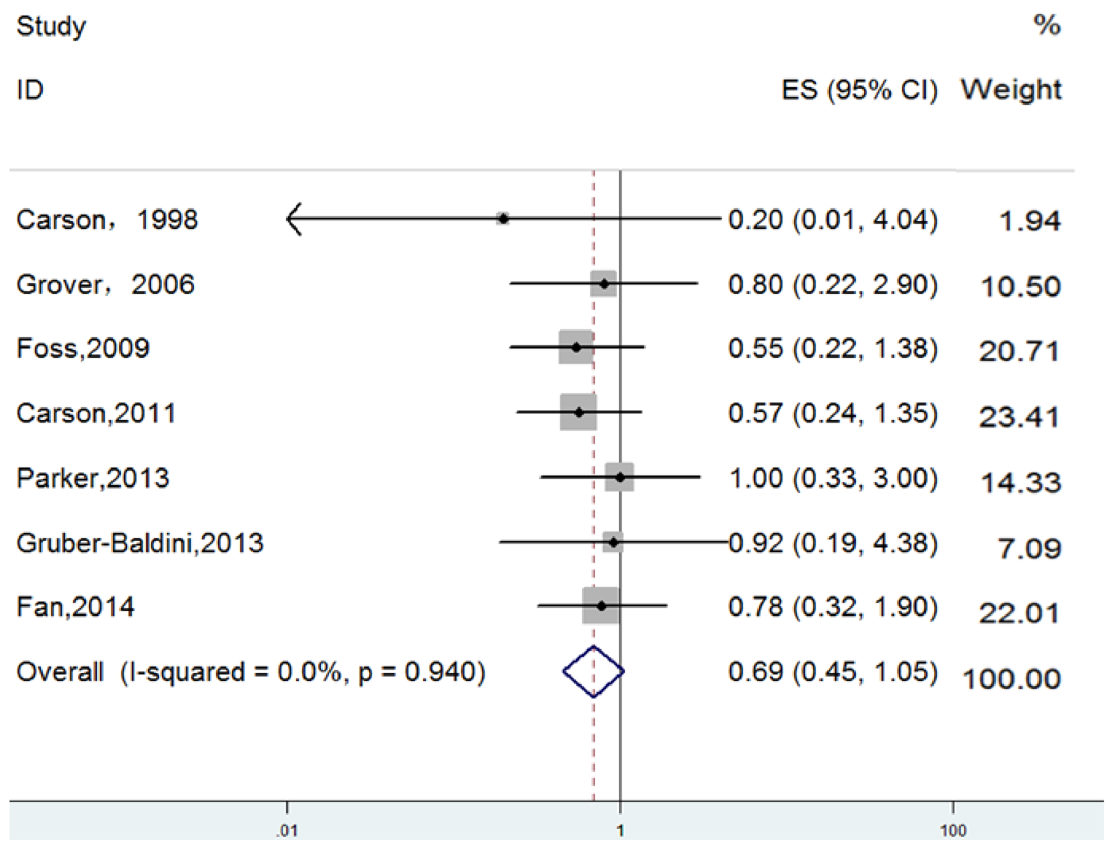

Supplementary Figure S5: Forest plot for RCTs after omitting So-Osman's trial in a fixed-effects model meta-analysis.
